# Degradation of the Molecular Basis of Life During the Aging Process

**DOI:** 10.3390/ijms27052419

**Published:** 2026-03-06

**Authors:** Janusz Wiesław Błaszczyk

**Affiliations:** Department of Human Behavior, Jerzy Kukuczka Academy of Physical Education, 40-065 Katowice, Poland; januszwblaszczyk@gmail.com

**Keywords:** life, aging, energy metabolism, glycolysis, morphogenesis, NAD

## Abstract

Aging is a chronic, destructive process characterized by the progressive breakdown of the body, leading to a loss of control over homeostasis. Glucose is the most important metabolite involved in metabolism and maintaining homeostasis in the human body. Glucose-based energy metabolism is fundamental to the activity and structural changes in the brain, which is the main regulator of life processes. Disturbances in energy metabolism and glucose-dependent metabolic processes have a decisive impact on the aging process. Age-related deficiency of the coenzyme NAD, which regulates glucose metabolism in neurons, leads to irreversible changes in the brain, culminating in senescence. Research on NAD precursors offers hope that although we cannot completely halt the aging process, NAD supplementation may enable healthy aging.

## 1. Aging

The phenomenon of an organism’s life can be described as a series of metabolic processes fueled by energy from the environment and regulated by signals that come from both the environment and within the organism itself [1]. Life is defined descriptively by the capacity for homeostasis, adaptation, metabolism, self-organization, growth, and reproduction [1]. All these are achieved by a set of self-organizing and self-sustaining processes, among which energy and information metabolism play a dominant role [1]. Energy and signaling metabolism are essential for the optimized interactions among cells, tissues, organs, and individuals. Together, these metabolic processes integrate the organism and give meaning to its existence.

Human personality is shaped through adaptation to the natural environment and the transmission of acquired traits—both genetic and epigenetic—to future generations [1]. These adaptive processes shape an individual’s phenotype, which encompasses not only physical characteristics but also biochemical and physiological traits, as well as behaviors that influence the overall lifespan of an organism. An individual’s lifespan is inherently limited by the time needed to achieve significant life goals. Most of these goals are typically achieved in the first half of life, encompassing development and adulthood. However, in the later phase of life, known as involution or aging, there is a gradual decline in all physiological adaptive mechanisms.

Recently, López-Otín and colleagues [2] presented twelve hallmarks of aging, including, among others, disturbances in homeostasis and energy metabolism, mitochondrial dysfunction, stem cell exhaustion, inefficient autophagy, impaired intercellular communication, and chronic inflammation. Generally, aging can be viewed as a vicious cycle of energy imbalances, resulting in physiological dysfunctions that further exacerbate the energy crisis in the aging body. This vicious cycle leads to inflammation, which causes a breakdown in vital energy metabolism. This chronic pathological state, known as “inflammaging” [3], leads to irreversible changes in cellular and systemic metabolism.

The development of age-dependent inflammation appears to be linked to impaired NAD+ homeostasis and a growing deficit in glucose-dependent energy metabolism. With age, metabolic deficiencies and increased cell death are accompanied by depleted macrophage-dependent phagocytosis, leading to chronic low-grade inflammation that promotes the development and progression of age-related metabolic diseases, which further accelerate the aging process.

The primary cause of inflammaging is a gradual shift away from optimal energy metabolism, leading to significant cell death that exceeds the body’s capacity for tissue repair. This process is accompanied by a reduction in signaling metabolism, which is essential for maintaining systemic functions. It is worth noting that glucose metabolism is a key link between energy metabolism and signaling in both physiological and inflammatory states. Thus, the tissues and organs with the highest energy consumption are the most susceptible to aging processes. This is especially true for the entire neuromusculoskeletal complex that forms the body’s motor system.

## 2. Metabolic Dysfunctions in Aging Cells

All life processes are fundamentally regulated by phosphate and calcium signaling [2,4]. The interaction between phosphate and calcium is essential for signaling and regulating important processes in the body [4,5]. As we age, disruptions in this interaction can lead to significant impairments in critical functions, highlighting the complexities of the aging process. The optimal coordination of energy metabolism depends on the mutual signaling among various participants, which ranges from the cellular to the systemic level. This multi-level communication is essential for signaling metabolism, enabling the coordination of activities across the gastrointestinal, respiratory, cardiovascular, and musculoskeletal systems, all under the direction of the nervous system.

At the cellular level, all life processes occur in a multistage metabolic pathway called cellular respiration, whose activity and efficiency are signaled by ATP levels. It is worth emphasizing that the extremely high level of ATP resynthesis during cellular oxidative phosphorylation indicates the importance of this metabolic process for life [6]. The energy invested in ATP during cellular respiration is then used to maintain the body’s resting metabolism and to carry out all motor behaviors. Therefore, energy obtained from the combustion of carbohydrates, especially glucose, can be converted into mechanical work during vital motor behaviors, relying on the coordinated activation of skeletal and smooth muscles, as well as the continuous work of the heart and diaphragm. ATP is released from muscle cells into the intercellular space as a result of mechanical deformation after muscle contraction. This ATP is then quickly broken down by ectonucleotidases [7]. Therefore, the main sources of ATP synthesis are organs operating under high mechanical load, particularly the heart and skeletal muscles [2,8]. In the heart, mitochondria account for about one-third of the cell’s volume, allowing for the continuous production of substantial amounts of ATP, mainly through the process of beta-oxidation [9].

Cardiac physiology plays a particular role in energy metabolism and the body’s life. Unlike most cells, cardiomyocytes primarily obtain their energy from chylomicrons, which are large lipoproteins rich in triglycerides produced in the enterocytes from dietary fats. These chylomicrons are transported directly to the heart via the lymphatic system. However, when present in excess, chylomicrons can enter the bloodstream and be stored in the liver. This accumulation of fatty acids is a major contributor to fatty liver disease, which gradually disrupts glucose metabolism throughout the body. To maintain optimal and life-sustaining levels of ATP production, the heart constantly consumes large amounts of energy, with fatty acids providing 60–90% of the energy [10].

## 3. Decline of Glucose Metabolism in Aging Cells

**Figure 1:**
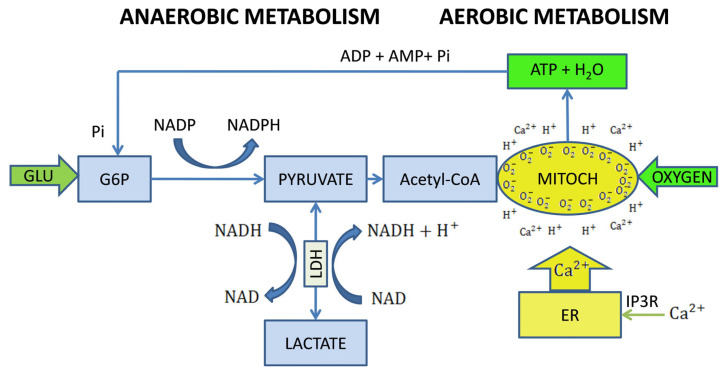
A simplified flowchart of glucose-based cellular energy metabolism, illustrating the critical points of anaerobic glycolysis and cellular respiration. At rest, cellular glucose uptake is determined by insulin-like growth factor-1 (IGF-1) activity and the level of phosphate derived from the hydrolysis of ATP produced by the heart and its own mitochondria (MITOCH). During states of increased cellular activity, the proportion of endogenous ATP increases rapidly, and its production is limited by calcium ions released from the endoplasmic reticulum (ER). Both organelles are characterized by high structural lability, requiring continuous reconstruction of their structure through anaerobic metabolic processes, in which the reversible conversion of lactate to pyruvate is catalyzed by lactate dehydrogenase (LDH) play fundamental role. An integral part of this process is the reduction of NAD+ to NADH, which provides electrons and proton flux used in oxidative phosphorylation (OXPHOS). The ATP produced by mitochondria is rapidly hydrolyzed in the cytoplasm, providing phosphates (Pi) and AMP, a sensitive indicator and regulator of the cell’s energy metabolism. The released Pi enables increased phosphorylation of glucose to G6P, which is essential for energy production, glycogen synthesis, and the pentose phosphate pathway. Intracellular NAD reserves are, however, used for energy production and for repair processes by NAD-consuming enzymes. Progressing NAD deficiency leads to a critical decline in energy metabolism, which is further exacerbated by inflammatory processes and culminates in aging and systemic disintegration.

The final measure of a cell’s vital activity and metabolic state is the amount of adenosine triphosphate (ATP) produced [11]. ATP is an unstable compound that quickly hydrolyzes into adenosine monophosphate (AMP) while releasing significant amounts of phosphate ions and functional groups. Consequently, AMP is regarded as a critical indicator and regulator of cellular energy metabolism (see Figure 1). The levels of ATP and, therefore, AMP primarily depend on the efficiency of two cellular metabolic pathways: glycolysis and cellular respiration. While cellular respiration accounts for the majority of ATP production, it relies entirely on the availability of oxygen and acetyl-CoA, which is produced from glucose during glycolysis [12].

ATP and its inorganic derivatives also play a fundamental role in the coordination of metabolic processes and the organismal signaling metabolism [4,5,7]. In most cells that utilize glucose as their primary energy fuel, glycolysis is the initial, rate-limiting step of cellular respiration [12,13]. Therefore, all intermediate glycolytic processes, particularly the biosynthesis of acetyl-CoA, which powers the cellular respiration pathway, ultimately depend on ATP levels. Consequently, the decline in oxidative phosphorylation in mitochondria that occurs with aging has a detrimental impact on cellular life and function.

Acetyl-CoA, the final product of glycolysis, plays a crucial role in cellular metabolism. It serves as fuel for cellular respiration, acts as a building block for fatty acids and lipids during lipogenesis, and functions as a donor for protein acetylation [14]. Additionally, acetyl-CoA is involved in various epigenetic processes that enable cells to differentiate and adapt to changing environments influenced by factors such as diet, stress, and toxins. Acetyl-CoA acts as a metabolic switch between lipolysis and cellular respiration. High levels of acetyl-CoA in the cytosol promote lipid synthesis, whereas lower levels shift metabolism toward cellular respiration [14]. Thus, acetyl-CoA is essential for the synergy between cellular metabolism and energy production. However, the excessive accumulation of acetyl-CoA can necessitate the action of deacetylase enzymes to remove nonenzymatic acetylation modifications that impair protein function. The sirtuin family of protein deacylases plays a vital role in repair and detoxification processes. In particular, the expression of SIRT3, an important protein located in the mitochondria, increases during fasting, in response to high-fat diets, and during exercise—conditions that lead to elevated acetyl-CoA levels in the mitochondria [14]. Sirtuins utilize the cofactor NAD+ to catalyze the process of protein deacetylation. However, a deficiency of NAD+ in aging cells hampers repair and detoxification processes, exacerbating age-related issues.

## 4. Impact of Age-Related NAD Deficiency on Cellular Energy Metabolism

The coenzyme nicotinamide adenine dinucleotide (NAD+) plays a crucial role in energy metabolism and cell signaling, functioning as both a donor and acceptor of electrons and protons during glycolysis. As a result, NAD-dependent intracellular processes significantly affect the efficiency of cellular energy metabolism, especially in the early stages of glycolysis. This reduction in efficiency is particularly noticeable in tissues that have high energy demands and rely heavily on glucose metabolism, such as the brain, muscles, and bones. Under normal physiological conditions, cells mainly replenish NAD through the salvage pathway, with only a limited contribution from tryptophan metabolism via the kynurenine pathway [15]. However, as we age, the efficiency of both pathways systematically decreases. Additionally, various chronic inflammatory conditions, particularly inflammaging, may further worsen this decline [16,17]. The decrease in NAD levels can be linked to glucose metabolism and the synthesis of NAD-consuming enzymes, such as sirtuins and poly(ADP-ribose) polymerases (PARPs) [18,19]. Consequently, NAD deficiency in aging cells hampers both energy metabolism and the functionality of NAD-dependent enzymes.

Intracellular NAD+ levels systematically decline with age [20], and this resulting NAD+ deficiency plays a key role in the aging process by limiting energy production, DNA repair, and genomic signaling [21]. Key NAD-consuming enzymes, such as poly(ADP-ribose) polymerase 1 (PARP-1) and sirtuins, are important in aging and cell death. Sirtuins regulate genome stability, stress response, autophagy, and apoptosis, and they are essential for the efficient differentiation of various cell types, including myoblasts, chondrocytes, endothelial cells, neural stem cells, mesenchymal stem cells, hematopoietic stem cells, and keratinocytes [22]. The decline in intracellular NAD+ levels with aging [20] has detrimental effects on cellular homeostasis and energy metabolism. This effect is first observed in tissues with high energy demands, such as the brain and skeletal muscles [16,19,23,24]. Several metabolic conditions—including cardiovascular disease, obesity, neurodegenerative diseases, cancer, and aging—have been linked to disturbances in intracellular NAD+ levels [25].

## 5. Deficiency of the Kynurenine Pathway

The kynurenine pathway is the primary biochemical pathway for the breakdown of tryptophan. It serves as the starting point for the de novo synthesis of nicotinamide adenine dinucleotide (NAD+), as well as for the production of serotonin and melatonin [26]. This pathway also functions as a feedback mechanism that helps suppress excessive immune responses, thereby contributing to the maintenance of homeostasis [27]. Under normal physiological conditions, the catabolism of tryptophan results in the formation of quinolinic acid. In the brain, quinolinic acid is a crucial precursor for NAD+ and acts as an agonist for neuronal NMDA receptors [28]. However, the toxicity of quinolinic acid arises from its ability to generate reactive oxygen species (ROS) and induce lipid peroxidation. Therefore, any dysregulation of kynurenine metabolites can be linked to neurological and psychiatric disorders, particularly in the elderly [29].

Inflammatory conditions, including those associated with aging, can increase the production of excitotoxins while simultaneously inhibiting the de novo synthesis of NAD+ [29]. Tryptophan metabolism plays a significant role in regulating the immune response [30,31]. Indoleamine-2,3-dioxygenase (IDO) is a key enzyme that catalyzes the first and rate-limiting step in the conversion of tryptophan to kynurenine and serves as a master regulator of the immune system, promoting immunosuppression to prevent excessive inflammation [32]. Notably, inflammation in peripheral tissues may lead to an accumulation of kynurenine in the brain, which is associated with disorders such as depression, schizophrenia, and neurodegenerative diseases [27,33].

## 6. Age-Related Changes in Calcium Signalling

The vicious cycle of energy dysmetabolism is influenced by various factors, including calcium metabolism. In cells with high energy demands—such as skeletal muscle, heart muscle cells (cardiomyocytes), and neurons—both primary and secondary calcium signaling play critical roles in vital functions and are also involved in apoptosis, acting as a life/death switch. Consequently, the intracellular accumulation of calcium ions due to age-related energy deficiencies leads to considerable cell death, further worsening the decline associated with aging. Oxidative stress, which increases with age, may also significantly contribute to accelerated aging of erythrocytes. Angiotensin II, which causes vasoconstriction, induces erythrocyte aging and increases susceptibility to cell death, partly because of heightened oxidative stress. Similar to necrosis, hemolytic erythrocytes lose the integrity of their cell membranes, leading to the release of their contents, which triggers an immune response and amplifies inflammation.

Calcitonin is a peptide hormone primarily produced by the thyroid gland. It plays a crucial role in regulating calcium and phosphate metabolism in the body by inhibiting the activity of osteoclasts in bones and increasing the excretion of these minerals through the kidneys [34]. This regulation of calcium and phosphate concentrations serves as a key stabilizing mechanism for metabolism, energy levels, growth, and body temperature. The main function of calcitonin is to prevent excessive or unwanted bone resorption. There is a gradual, age-related decrease in circulating calcitonin levels in both men and women. In young adults, calcitonin secretion is stimulated by estrogens, and a significant deficiency in calcitonin could contribute to the development of post-menopausal bone loss due to excessive bone resorption. This loss of control over calcium signaling can significantly accelerate the aging process. In myocytes, cardiomyocytes, and neurons, increased calcium inflow may hinder oxidative phosphorylation by disrupting the synergy between calcium ions and protons [35]. This disruption can lead to substantial cell death through apoptosis.

Locomotion and cyclical mechanical loading are crucial in regulating the release of calcium ions (Ca^2+^) and various signaling molecules. Notably, insulin-like growth factor-1 (IGF-1) and β-catenin are vital for the survival of osteocytes and promote anabolic effects. Osteocytes also secrete fibroblast growth factor 23 (FGF23), which helps regulate serum phosphate levels by acting on the kidneys, heart, and parathyroid glands [3]. Transient increases in intracellular calcium ions facilitate local adjustments to the cytoskeleton. However, sustained high levels of intracellular Ca^2+^ can be harmful, disrupting cytoskeletal components, including actin filaments [36]. Osteocyte apoptosis is a significant risk factor linked to aging, reduced physical activity, hormone deficiencies, and inflammation, all of which lead to a notable decrease in osteocyte density [37].

Osteocytes are essential for regulating both skeletal and systemic homeostasis by closely interacting with immune cells, adipocytes, and hematopoietic cells in the bone marrow. Their functions are influenced by the hypothalamus and the pituitary gland [38]. During apoptosis, osteocytes send signals to osteoclasts, which then recruit them to sites of bone resorption. The accumulation of apoptotic osteocytes with aging is associated with osteonecrosis and osteoporosis, increasing the risk of fractures.

Osteocalcin is a protein produced by osteoblasts, primarily during locomotory movements. It plays a crucial role in regulating bone metabolism by promoting bone mineralization. Additionally, osteocalcin also acts as a signaling molecule, a hormone that influences glucose and fat metabolism. It increases insulin secretion, improves insulin sensitivity, and helps regulate blood glucose levels. Osteocalcin concentrations in the blood tend to decline with age, and human studies indicate that lower levels of osteocalcin in the blood are associated with poorer cognitive performance in older adults [39].

## 7. Emotional Stress and Aging

Any systemic energy deficit is signaled by stress. In older individuals, disturbances in glucose metabolism can lead to chronic stress and heightened anxiety, significantly impacting the aging process. Prolonged elevated levels of cortisol and adrenaline, resulting from both physical and emotional stress, promote the activity and differentiation of osteoclasts—cells responsible for breaking down bone—while inhibiting the bone-forming activity of osteoblasts. This imbalance can lead to significant bone loss, known as osteoporosis, and disrupt calcium metabolism and signaling. As a result, it may impair muscular activity and lead to progressive skeletal muscle atrophy [34]. The combination of age-related changes, a decline in energy metabolism, elevated calcium activity, and decreased ATP production can be particularly detrimental and even life-threatening for the elderly.

During sleep, when blood glucose levels drop below a certain threshold, the stress hormone cortisol is released into the bloodstream. This secretion interrupts sleep, motivating us to eat to restore normal blood sugar levels. With age, hepatic glucose metabolism, responsible for maintaining the energy metabolism of most tissues, declines [40]. This results in an increase in pathological processes related to glycotoxicity [41,42] and lipotoxicity [43]. Their pathogenic mechanisms include oxidative stress, the production of reactive oxygen species, cell apoptosis, and mitochondrial damage. Tissue and organ damage progresses with age, leading to, among other things, pancreatic cell dysfunction, increasing insulin resistance, and the development of diabetic complications. Furthermore, the liver’s function in glucose storage exacerbates fatty liver disease. All of this leads to increased stress responses in the brain, increased anxiety, and sleep disturbances in older adults.

One of the physiological purposes of sleep is to remove toxic metabolic byproducts that accumulate in the brain during daily activities [44]. Consequently, chronic sleep disturbances in older age disrupt the glymphatic system, causing an accumulation of toxic byproducts resulting from neuronal metabolism [4]. In particular, the buildup of proteins such as beta-amyloid and hyperphosphorylated tau can result in significant cell death in affected brain regions, ultimately leading to functional dysfunction in those areas.

Stress hormones directly impact the gut, disrupting motility, mucus production, and immune responses, allowing bacteria and toxins to penetrate the gut lining. Chronic stress, in particular, significantly disrupts the gut microbiome, causing dysbiosis, a weakened gut barrier, and impaired gut function. It also impacts the gut–brain axis and contributes to inflammation and various gastrointestinal and mood disorders. Age-related changes in the gut may include mucosal damage, impaired nutrient and electrolyte absorption, breakdown of the gut barrier, increased immune cell infiltration, motility disorders, dysbiosis, dysfunction of enteroendocrine cells, and disruptions in the enteric and autonomic nervous systems. The gut–brain axis is a powerful, bidirectional communication network that connects the central nervous system with the gastrointestinal tract through the vagus nerve, hormones, immune signals, and neurotransmitters such as serotonin, dopamine, and gamma-aminobutyric acid (GABA) [45,46,47,48]. This axis influences mood, cognition, and stress, which helps explain how gut dysbiosis—imbalances in gut microbiota—can impact mental health. Sialic acid Neu5Ac is crucial for shaping adult microbiota and influencing brain function. These changes can contribute to neuroinflammation and neurodegeneration. Importantly, during the aging process, chronic stress can make both the intestinal epithelium and the blood–brain barrier more permeable and less selective. This increased permeability heightens the risk of mental and neurological problems in older adults [49].

Recent research has shown that motor behaviors can have a positive impact on brain function, improving attention, memory, neurogenesis, and reward system functioning [50]. One potential mediator of these benefits is irisin, a hormone secreted during physical activity by skeletal muscle, the heart, and subcutaneous fat, with some contribution from the liver. Irisin plays a crucial role in regulating energy metabolism, glucose metabolism, the browning of adipose tissue, and various other physiological processes [51]. Notably, irisin can cross the blood–brain barrier, helping to reduce neurodegeneration and neuroinflammation [50]. Irisin deficiency is often observed in hypoactive elderly individuals [52] and in patients with Parkinson’s disease [53]. Therefore, increasing motor activity in older adults is recommended, as it may help alleviate symptoms of stress and depression. Additionally, enhanced skeletal muscle activity appears to prevent the buildup of kynurenine toxins in the body, which are known to have a detrimental impact on mental health [27].

## 8. Impact of Aging on the Motor Behavior

Active movement is a fundamental expression of life in the body. The body’s main metabolic system is responsible for converting energy from food into mechanical work performed by the heart, diaphragm, and both smooth and skeletal muscles. Maintaining an optimal and safe balance between energy intake and expenditure is a crucial challenge for all living organisms. Consequently, the processes that consume energy, such as physical exertion, are tightly regulated. Information about the body’s current energy fuel status—primarily glucose in humans—is quickly relayed to the brain, where it influences motivation and emotional responses.

Human locomotory movements and nearly all forms of physical exercise are dependent on skeletal muscle activity and their significant energy consumption. Glucose serves as the primary energy fuel for the human body. At rest, the body stores most of its glucose in skeletal muscle and the liver. In a young, healthy individual, approximately 100 g of glucose is stored as glycogen in the liver, while nearly 400 g is stored in skeletal muscle. Under normal physiological conditions, muscle glycogen is the main source of energy for muscles. During exercise, when glucose utilization increases tenfold, the body maintains a constant blood glucose level by utilizing liver glycogen. As people age and physical activity declines, the ability of skeletal muscle to store glucose is impaired. This decline results from the gradual loss of muscle mass with age and the accumulation of glycogen in muscles, which can become toxic. Disturbances in glucose metabolism in older individuals can lead to chronic elevations in blood glucose levels, a condition known as “glucose toxicity.” This toxicity can contribute to pancreatic beta-cell dysfunction and is considered a complication of type 2 diabetes.

## 9. Balance Between Recycling and De Novo Processes in Aging Cells

Protein and lipid recycling processes—the fundamental structural components of phospholipid membranes—are crucial for maintaining vital processes and cellular homeostasis. These intracellular pathways are coordinated and regulated by various byproducts, including AMP and acetyl-CoA. The main signal of the metabolic state of the cell is AMP [14]. An increase in AMP levels enhances the activity of the protein kinase AMPK, which controls protein phosphorylation, inhibits lipogenesis, and enhances fatty acid oxidation in tissues. AMP regulates the activity of AMP-activated protein kinase (AMPK), which phosphorylates various proteins involved in maintaining cellular energy balance. Under conditions of sufficient nutrient excess, AMPK initiates autophagy, while mammalian target of rapamycin kinase (mTOR) inhibits it [17].

The recycling of glycoproteins and glycolipids is crucial for cells to maintain homeostasis and respond to environmental stimuli; however, these processes consistently decline, which is one of the main causes of cell aging. Particularly, in long-lived cell populations that do not undergo cellular turnover, such as neurons, cardiomyocytes, and osteocytes, the efficiency of glycoprotein and glycolipid metabolism plays a critical role in determining cell, tissue, and organismal viability.

Another important metabolic pathway related to glucose is the hexosamine biosynthesis pathway. This pathway regulates various post-translational modifications, including glycosylation, phosphorylation, acetylation, ubiquitination, and hydroxylation [54]. Glycosylation plays a key role in maintaining metabolic homeostasis, influencing stem cell biology, affecting signaling pathways, regulating transcription factor function, and supporting immune cell maintenance [54]. Glycosylation is the most common protein modification in long-lived cells. This process of adding complex sugars to adhesion proteins is essential for maintaining cellular function by recycling worn-out membranes and organelles. It primarily takes place in lysosomes, the endoplasmic reticulum, and the Golgi apparatus [55]. The impairment of these processes that progresses with age is another aging mechanism.

Cellular organelles, especially lysosomes, the Golgi apparatus, and mitochondria, play active roles in energy-driven cellular metabolism (see Figure 1). Mitochondria act as cellular energy systems, continuously being rebuilt and adjusted according to the levels of cellular metabolism. It is important to understand that the carbohydrates present in glycoconjugates are primarily derived from glucose, with glycolysis being crucial for both cellular respiration and lipogenesis [12,13]. Lipogenesis and protein sialylation significantly influence morphogenesis and enhance cellular and tissue function. Cell membranes and organelles are composed of glycoproteins and glycolipids, which greatly affect the function and longevity of tissue structures. Research in animals has shown that glycoproteins have a turnover rate that can vary from hours to days, while AMPA glutamate receptors can remain functional for up to two days [55].

## 10. Senescent Changes in Mitochondria

The dynamic process of mitochondrial turnover is essential for optimizing cellular energy metabolism. This process allows for the adjustment of the number, position, and shape of mitochondria according to current energy demands. It achieves this balance through mitochondrial biogenesis (the creation of new mitochondria) and mitophagy (the removal of damaged mitochondria), which work together to maintain energy homeostasis. The turnover of the inner mitochondrial membrane, which is rich in proteins, is particularly important for the energy metabolism of neurons and affects their cellular lifespan [56,57]. Disruptions in these complex processes can lead to mitochondrial dysfunction, which significantly contributes to the aging process [58].

Mitochondria adapt dynamically to a cell’s energy demands by altering their morphology and function, engaging in active interactions with other cellular organelles and the cytoskeleton [59]. Specifically, the interactions between actin and mitochondria are vital for regulating mitochondrial turnover. Actin can influence mitochondrial dynamics by modulating fission and fusion at mitochondrial-endoplasmic reticulum contacts and by facilitating the transport of mitochondria along actin filaments. Actin filaments are continuously assembled and disassembled, driven by ATP hydrolysis [60]. Thus, in senescence cells, which exhibit deficient respiration and ATP production, actin-dependent processes are significantly impaired.

To maintain mitochondrial dynamics, AMPK induces cytoskeletal reorganization, specifically increased actin production and microtubule network formation [61]. Microtubule networks are dynamic intracellular structures that play a crucial role in maintaining cell shape, facilitating intracellular transport, promoting cell division, and enabling cell movement [62]. They must constantly reorganize to adapt to the cell’s needs. Microtubules participate in the functioning of mitochondria, the Golgi apparatus, and the endoplasmic reticulum. Dynamic microtubule instability causes the continuous and rapid turnover of most microtubules, which have a half-life of only a few minutes [63].

A crucial aspect of mitochondrial function is the limited production of reactive oxygen species (ROS) [54]. When mitochondria malfunction, cells become more susceptible to pro-inflammatory cytokines and activated inflammatory pathways, leading to excessive ROS production and subsequent cellular damage [64,65]. During an anti-inflammatory response, activated macrophages produce tumor necrosis factor (TNF-α), which inhibits mitochondrial complex I, a key component responsible for electron and proton transport, resulting in cellular death [66].

Mitochondrial dysfunction and impaired energy metabolism are key features of neurodegenerative diseases, attributed to reduced expression or activity of peroxisome proliferator-activated receptor gamma coactivator 1-alpha (PGC1-α). This reduction leads to deficits in mitochondrial dynamics [67]. PGC-1α is highly expressed in tissues with significant oxidative metabolism, such as skeletal muscle, brain, heart, and brown adipose tissue [68]. Lactate dehydrogenase is an essential enzyme that facilitates the conversion between pyruvate and lactate, alongside interconverting the cofactors NADH and NAD+. These conversions are the primary source of protons (H+) necessary for maintaining the acidic environment of lysosomes and for executing oxidative phosphorylation in mitochondria (see Figure 1). The activity of lactate dehydrogenase is regulated by PGC-1α in response to various environmental and intracellular signals, with modulation from AMPK, sirtuins, and mitochondrial transcription factors [17]. PGC-1α adjusts cellular energy metabolism according to current needs by enhancing mitochondrial dynamics, promoting oxidative phosphorylation, and detoxifying reactive oxygen species [69,70]. It also plays a role in the co-regulation of lipid and carbohydrate metabolism through processes such as fatty acid β-oxidation and gluconeogenesis.

Physical activity and energy stress stimuli, including hypoglycemia, increase PGC-1α expression in various energy metabolism-related tissues [70,71]. AMP-activated protein kinase (AMPK) primarily activates PGC-1α through phosphorylation [17,70]. A deficiency in PGC-1α leads to increased mitochondrial dysfunction, which is particularly evident in aging and neurodegenerative diseases [70]. Molecular pathways regulated by PGC-1α connect oxidative stress and mitochondrial metabolism to the inflammatory response and metabolic syndrome [70]. A deficiency in PGC-1α is linked to the development of metabolic syndrome, characterized by chronic, low-grade inflammation. These pathogenic processes can result in conditions such as obesity, type 2 diabetes, cardiovascular disease, and fatty liver disease. Low levels of PGC-1α, accompanied by inflammation, downregulate the expression of antioxidant genes in mitochondria, induce oxidative stress, and activate nuclear factor kappa B [70].

## 11. Aging Lysosomes

Lysosomes are also highly dynamic organelles that can quickly change their position, number, shape, size, composition, and activity in response to environmental and cellular signals. They adapt to the current needs of the cell through various processes that occur within minutes to hours [72]. Lysosomes play a vital role in essential metabolic processes, including the degradation and recycling of proteins and lipids, waste removal, membrane repair, and the regulation of energy metabolism [72]. Lysosomal exocytosis is particularly important for repairing the cell membrane. As the final organelles in the endocytic pathway, lysosomes degrade macromolecules and process their components into nutrients for the cell [73]. An age-related decline in lysosomal activity leads to an irreversible accumulation of undegraded substrates, resulting in various clinical symptoms depending on the specific substrate and its location of accumulation [74].

Lysosomes function optimally due to their unique lipid membrane structure, which is characterized by a thick glycocalyx layer. This structure enables them to maintain a highly acidic pH of around 5 in their lumen [72]. Under normal physiological conditions, the vacuolar ATPase utilizes energy from ATP hydrolysis to sustain a high concentration of protons. However, both the glycocalyx structure and the functions of lysosomes gradually deteriorate with aging, resulting in a decline in energy metabolism [72]. In older cells, reduced cellular respiration leads to decreased ATP production, which results in long-lasting impairments in lysosomal function. Additionally, as we age, disturbances in glucose-based energy metabolism can worsen, negatively affecting lipogenesis and altering the quality and function of mitochondria and cell membranes. The accumulation of these dysfunctions is the primary cause of tissue and organ deterioration, a phenomenon commonly referred to as aging [72].

## 12. Dysmorphic Changes in Senescent Tissues

Insufficient energy metabolism negatively impacts all cellular biochemical processes, including morphogenesis, and the associated buildup of abnormal proteins and lipids accelerates the aging process [75]. Morphogenesis is a vital process that determines the size, shape, and cellular composition of tissues and organs, ultimately influencing their physiological functions. Chronic glycation, observed in aging cells, is a typical example of a pathomechanism that causes structural damage, decreases tissue elasticity, and accelerates the aging process.

The glycocalyx, a protective outer layer surrounding cell membranes, consists of glycosaminoglycans, proteoglycans, and various glycoproteins, including acidic oligosaccharides and sialic acid—specifically N-acetylneuraminic acid (Neu5Ac) [76,77]. This human-specific sialic acid is essential for the proper functioning of cell membranes, organelles, and the glycocalyx in all body cells. It imparts unique biomechanical and physicochemical properties to tissues and organs, providing them with the necessary elasticity and permeability for physiological function.

The glycocalyx forms a functional barrier between the cell and its environment, facilitating intercellular interactions and protecting the cell membrane from mechanical stress. This barrier allows maintain cell shape and tissue integrity [77]. Additionally, the extracellular matrix, a dynamic network of proteins and polysaccharides surrounding cells, offers a physical scaffold and regulates various cellular repair processes, including stem cell migration and differentiation, morphogenesis, and thus contributes to homeostasis [78]. The delicate structures of the extracellular matrix and glycocalyx, however, are particularly fragile to changes in structural composition and energy metabolism. As a result, they undergo continuous remodeling, making them vulnerable to age-related changes.

In blood vessels, the endothelial glycocalyx acts as a barrier, separating the vessel wall from circulating erythrocytes. The endothelial glycocalyx in the brain is a structural component of the blood–brain barrier, creating the primary interface between blood and cerebral blood vessels [59,79,80]. The endothelial glycocalyx participates in controlling inflammatory responses, including leukocyte turnover and the extravasation of pathogens such as bacteria, viruses, and cancer cells [81]. The surface of the vascular endothelium is particularly abundant in sialic acid, which provides electrostatic repulsion that inhibits erythrocyte adhesion and promotes their smooth flow throughout the circulatory system [79,82].

In aging or inflammatory conditions, the endothelial glycocalyx can sustain damage, resulting in increased vascular permeability, tissue edema, heightened leukocyte adhesion, platelet aggregation, and impaired vasodilation. Because it is constantly exposed to friction forces and changes in inflammatory processes, the endothelial glycocalyx must undergo continuous regeneration, while a deficiency in this process has been linked to various pathologies, including atherosclerosis, hypertension, vascular aging, and vascular diabetes [81].

The lymphatic vascular system is essential for fluid transportation, immune reaction, and lipid absorption [83]. The lymphatic vasculature provides a unidirectional conduit that returns filtered interstitial fluid and tissue metabolites to the blood circulation [83,84]. Cellular dysmorphism particularly strikes the vascular system. While blood vessels are essential for oxygen and nutrient delivery, as well as the disposal of waste products for detoxification and replenishment, the lymphatic vasculature plays crucial roles in immune surveillance, lipid absorption, and the maintenance of tissue fluid balance [83]. Lymphatic vessel dysfunction has been associated mainly with chronic tissue inflammation caused by lymphatic stasis, resulting in damaged or incompetent lymphatic vessels. Lymphatic vascular defects have been discovered in conditions including obesity, cardiovascular disease, inflammation, hypertension, atherosclerosis, Crohn’s disease, glaucoma, and several neurological disorders.

Pathological calcification of cardiovascular structures is a common complication associated with aging [85,86]. In older individuals, high blood levels of phosphates and calcium often coincide with declining levels of ATP, primarily due to reduced mobility. ATP is essential as it directly inhibits vascular calcification. A decrease in ATP leads to a deficiency of pyrophosphate, an inorganic anion composed of two phosphorus atoms connected by an oxygen atom. Pyrophosphate is typically produced as a metabolic byproduct during ATP hydrolysis [86].

Ectonucleotide pyrophosphatase/phosphodiesterase I (ENPP1) is a type II transmembrane glycoprotein located on plasma membranes and ER lumen with nucleotide pyrophosphatase and phosphodiesterase enzymatic activities, critical for calcium and purinergic signaling [87]. Specifically, purinergic signaling is involved in platelet aggregation, muscle contraction, cell proliferation, migration, differentiation, and apoptosis, as well as in regulating hypoxia and ischemia in tissues [87]. Many disorders related to mineralization, calcium metabolism, or calcification have been associated with loss-of-function mutations in the ENPP1 gene [87]. ENPP1 plays a regulatory function in immune cells such as neutrophils, macrophages, dendritic cells, natural killer cells, and B lymphocytes.

Human cells can synthesize and utilize only Neu5Ac [88]. Therefore, consumption of red meat and dairy products containing high concentrations of N-glycolylneuraminic acid (Neu5Gc), a foreign protein, can induce inflammation, disease processes, and accelerated aging [89]. Neu5Gc, when ingested, integrates into human glycans as a rare “xeno-autoantigen,” potentially triggering chronic inflammation linked to atherosclerosis and cancer [89]. Neu5Gc is found in endothelial tissues of atherosclerotic plaques and is linked to cardiovascular diseases and various cancers [79,82,89]. Hypersialylation, or changes in sialic acid levels, is commonly seen in cancer cells, where a thick layer of sialoglycans allows them evade immune detection [79].

Carnosine acts as an antiglycating agent and helps to neutralize reactive oxygen species (ROS) and alpha-beta-unsaturated aldehydes that result from fatty acid peroxidation in cell membranes during oxidative stress. This role is important as it limits the formation of atherosclerotic plaques. Although the body naturally produces carnosine in the liver, its levels in the blood, brain, heart, and skeletal muscles decline with age [90]. The reduction in carnosine levels may contribute to the onset or progression of various degenerative diseases, including diabetes, atherosclerosis, chronic renal failure, and neurodegeneration. Therefore, carnosine supplementation may help slow the aging process due to its antiglycating properties [90].

## 13. Can We Stop the Aging Process?

Systemic aging is an irreversible process characterized by the decline of the body’s organs and tissues due to disturbances in energy metabolism, leading to a loss of homeostasis and allostasis. Metabolic dysfunction begins at the cellular level, gradually affecting tissue and organ function and their interactions. The specific dynamics of aging vary among tissues, depending on their anatomical structure and the organ’s role in vital processes. The leading role in the aging process is played by the brain, which controls and coordinates all metabolic processes at all levels, from the cellular to the systemic level, and importantly, its tissue structure is subject to progressive and irreversible degradation.

Early signs often involve disorders in glucose metabolism, particularly in organs with high energy demands, such as the brain, heart, and muscles. These organs’ cells show increased activity in cellular structures, and dysfunctions in mitochondria and lysosomes are linked to aging. The synergy between glucose and lipids is crucial, especially for neuromuscular and skeletal systems, which can exhibit noticeable changes in motor behavior due to impaired energy metabolism. The fundamental mechanisms of aging discussed in this work highlight the complexity and irreversibility of these processes. This insight urges us to enhance scientific research into the still unclear aspects of aging and, most importantly, to explore potential therapies that can slow down the aging process. Such advancements would significantly improve the quality of life for the rapidly growing elderly population.

Therapy aimed at halting the decline of nicotinamide adenine dinucleotide (NAD+) appears to be very promising at this time. As mentioned earlier, NAD+ is a crucial cofactor in many essential cellular processes, including energy metabolism. Currently, nicotinamide mononucleotide (NMN) and nicotinamide riboside (NR) are tested to treat the age-related NAD+ deficiency [91,92,93,94,95]. The supplementation of NAD precursors has been shown to improve NAD+ biosynthesis in various peripheral tissues, including the pancreas, liver, adipose tissue, heart, skeletal muscle, kidney, testis, eyes, and blood vessels, under both normal and pathophysiological conditions [92]. NAD supplements can cross the blood–brain barrier, increasing NAD+ levels in brain areas such as the hippocampus and hypothalamus [92]. The pancreatic β-cells also positively respond to NAD supplements by increasing glucose-stimulated insulin secretion, thereby improving glucose tolerance [92]. Supplementation with NAD precursors helps alleviate hepatic insulin resistance induced by a high-fat diet by restoring NAD+ biosynthesis, enhancing SIRT1 activity, and promoting gene expression related to inflammation, oxidative stress, and circadian rhythms [92]. Long-term NR or MNM supplementation can reduce age-related inflammation in adipose tissue and improve overall insulin sensitivity, independent of any changes in body weight [92]. Additionally, NMN boosts SIRT3 activity and promotes lipid oxidation in the liver’s mitochondria [92]. Oral NR supplementation can mitigate diabetic neuropathy [91].

The decrease in NAD+ levels observed with aging negatively impacts all stages of neuronal respiration, which are vital factors in the aging process. Therefore, supplementation with NAD+ precursors, particularly nicotinamide riboside (NR) and nicotinamide mononucleotide (NMN), restores physiological NAD+ levels, quite effectively alleviates metabolic syndrome, improves cardiovascular and muscular function, and slows neurodegeneration [93,94,95]. Oral administration of NAD+ precursors represents a potential strategy for reversing the physiological impairments associated with aging. Supplementation with NMN and NR seems to protect against type 2 diabetes, Alzheimer’s disease, endothelial dysfunction, inflammation, and gut dysbiosis, among other conditions [93,94,95]. Overall, NAD+ therapies appear to have beneficial physiological effects by slowing age-related metabolic dysfunction. However, further research is needed to fully explore the effects of this therapy.

## Data Availability

No new data were created or analyzed in this study. Data sharing is not applicable to this article.
